# Association Between Residual Inhibition and Neural Activity in Patients with Tinnitus: Protocol for a Controlled Within- and Between-Subject Comparison Study

**DOI:** 10.2196/12270

**Published:** 2019-01-09

**Authors:** Suyi Hu, Lukas Anschuetz, Markus E Huth, Raphael Sznitman, Daniela Blaser, Martin Kompis, Deborah A Hall, Marco Caversaccio, Wilhelm Wimmer

**Affiliations:** 1 Hearing Research Laboratory ARTORG Center for Biomedical Engineering Research University of Bern Bern Switzerland; 2 Department of Ears, Nose, Throat, Head and Neck Surgery Inselspital, Bern University Hospital University of Bern Bern Switzerland; 3 Ophthalmic Technology Laboratory ARTORG Center for Biomedical Engineering Research University of Bern Bern Switzerland; 4 National Institute for Health Research Nottingham Biomedical Research Centre University of Nottingham Nottingham United Kingdom; 5 Hearing Sciences, Division of Clinical Neuroscience School of Medicine University of Nottingham Nottingham United Kingdom; 6 Nottingham University Hospitals National Health Service Trust Queens Medical Centre Nottingham United Kingdom; 7 Malaysia Campus University of Nottingham Semeniyh Malaysia

**Keywords:** electroencephalography, heterogeneity, high-frequency audiometry, neural activity, tinnitus, tinnitometry

## Abstract

**Background:**

Electroencephalography (EEG) studies indicate possible associations between tinnitus and changes in the neural activity. However, inconsistent results require further investigation to better understand such heterogeneity and inform the interpretation of previous findings.

**Objective:**

This study aims to investigate the feasibility of EEG measurements as an objective indicator for the identification of tinnitus-associated neural activities.

**Methods:**

To reduce heterogeneity, participants served as their own control using residual inhibition (RI) to modulate the tinnitus perception in a within-subject EEG study design with a tinnitus group. In addition, comparison with a nontinnitus control group allowed for a between-subjects comparison. We will apply RI stimulation to generate tinnitus and nontinnitus conditions in the same subject. Furthermore, high-frequency audiometry (up to 13 kHz) and tinnitometry will be performed.

**Results:**

This work was funded by the Infrastructure Grant of the University of Bern, Bern, Switzerland and Bernafon AG, Bern, Switzerland. Enrollment for the study described in this protocol commenced in February 2018. Data analysis is currently under way and the first results are expected to be submitted for publication in 2019.

**Conclusions:**

This study design helps in comparing the neural activity between conditions in the same individual, thereby addressing a notable limitation of previous EEG tinnitus studies. In addition, the high-frequency assessment will help to analyze and classify tinnitus symptoms beyond the conventional clinical standard.

**International Registered Report Identifier (IRRID):**

RR1-10.2196/12270

## Introduction

### Background

About 10%-15% of the general population is suffering from chronic subjective tinnitus, that is, consciously perceiving sound without the presence of physical sound sources [1]. Tinnitus can lead to health problems, distress, and psychological complaints and can substantially impair the quality of life [2]. In most cases, tinnitus occurs after cochlear damage such as sensorineural hearing loss [3], presbycusis [4], excessive noise exposure, and noise trauma [5]. Chronic tinnitus is also experienced by people with otherwise normal hearing [6]. Furthermore, tinnitus can be generated by head and neck injury, infections and neck illness, drugs, and other medical conditions [5] and may also be influenced by emotional and mental conditions [7]. Extensive causes and symptoms could complicate the diagnosis and treatment of tinnitus; to improve this, studies have proposed classifying tinnitus into different subtypes according to symptoms such as perceptual characteristics, laterality, loudness, or symptom severity, as well as the presence of tinnitus-associated disorders [8-10]. Alternatively, the categorization of tinnitus has been based on the response to applied treatments [11]. Nevertheless, an incomplete understanding of the underlying pathophysiology and the presence of numerous psychosocial and environmental factors that could influence intervention results may cause heterogeneity and preclude tinnitus subgrouping. As an effect, to date, there are no singularly effective clinical evaluation and treatment methods for subjective tinnitus [12]. Moreover, the current tinnitus diagnosis heavily relies on patient-reported assessments such as questionnaires. Currently, more objective assessment methods, which could provide physiology-based measures of comparison, do not exist.

As tinnitus often originates from peripheral and central auditory mechanisms [13,14], the assessment of abnormal neural activity may be a potential approach for objective diagnosis. A number of research groups have suggested that tinnitus is accompanied by changes in the brain [15-20] and can be examined using electroencephalography/magnetoencephalography (EEG/MEG) [17,19,21-27]. In particular, spontaneous brain oscillations, that is, the ongoing brain activity in the absence of external events, have been intensively investigated using EEG/MEG. Traditional EEG/MEG power bands of resting-state activity have been quantified between people with tinnitus and controls [17,22]. One theory explaining group differences in the EEG/MEG power within specific frequency bands focuses on the thalamocortical dysrhythmia model. This model predicts an increase of power in low frequencies (delta and theta bands) and high frequencies (gamma band) in the auditory cortex in subjects with tinnitus [18]. Furthermore, marked alterations of EEG/MEG oscillations across other brain regions and power bands have been reported in the prefrontal cortex [28-31], cingulate cortex [29-38], parahippocampus [15,26,31,34,36,37,39-42], and insula [26,37,43], implying the involvement of other brain areas and power bands. Nevertheless, poor matching of gender, age, and hearing loss status across groups, as well as other confounding factors, hinder the interpretation of study findings and generate inconsistencies [44]. To minimize variance across subjects, a within-subject measurement comparing the brain activity during a baseline period (tinnitus with abnormal brain activity) with a period after the suppression of tinnitus (stabilization of abnormal activity) may overcome this limitation.

Residual inhibition (RI) is a temporary forward tinnitus suppression mechanism, which can reduce or alleviate tinnitus loudness for a short duration after the presentation of an acoustic stimulus [45,46]. Previous work has compared the brain activity between baseline (tinnitus) and poststimulus (reduced tinnitus or nontinnitus) periods on the group level, observing reduced power in the delta frequency band in the temporal region after stimulus exposure, which is in accordance with the thalamocortical dysrhythmia [47]. In a different study, neural activity changes between pre- and poststimulus periods were detected in nonauditory cortices, that is, in the left anterior superior temporal gyrus, the motor cortex, and the posteromedial cortex by a single subject with musical hallucination using music as suppression stimulus [48]. Adjamian et al compared the neural activity during masking and resting state [24]. Sedley et al detected changes in the tinnitus-related neural activity on a single-subject level, indicating the potential for within-subject comparisons to minimize data heterogeneity [49]. Moreover, RI has been investigated with high-precision recordings of the neural activity by using intracranial monitoring of a single subject, in which tinnitus-linked, low-frequency neural oscillations were observed in auditory cortical regions, as well as other brain areas, and interacted with the middle- and high-frequency activity [50]. Reportedly, the distinct response of the neural activity to control stimuli that do not induce RI indicated that these changes correlated with RI [47,49]. Nevertheless, no comparison was performed with nontinnitus subjects perceiving RI stimuli, which could further demonstrate the correlation between RI and the tinnitus-associated neural activity.

This study aims to examine the brain activity in tinnitus and nontinnitus subjects using whole-scalp EEG recordings. RI will be used to modulate the tinnitus perception and enable a within-subject comparison. In addition, the RI stimuli will be matched with the tinnitus frequency of each participant, while the same low-frequency (0.5 kHz) control stimuli will be presented to all participants to exclude any effects of nonspecific sound-induced responses. We extend the applicable frequency range up to 13 kHz to enable a more accurate frequency identification of tinnitus and RI characteristics across a wider portion of the audibility range. Moreover, including nontinnitus subjects enables to compare between matched tinnitus and nontinnitus groups (ie, hearing loss characteristics) to determine whether the observed differences in the neuronal activity after RI are tinnitus-specific.

### Objectives

The primary objective of this study is to identify tinnitus-associated neural oscillations in resting-state (spontaneous) scalp EEG data. We hypothesize that differences due to RI in the power spectral density (PSD) of baseline (tinnitus) and poststimulus (reduced tinnitus) measurements within the same subjects can be detected in traditional EEG power bands. The secondary objectives of this project are as follows; (1) to improve understanding of the RI effect on the tinnitus-associated neural activity; (2) to identify differences in the PSD of the baseline period between the tinnitus and control groups; (3) to identify differences in the PSD of the poststimulus period between the tinnitus and control groups; and (4) to evaluate whether within-subject EEG data collection can reduce heterogeneity.

## Methods

### Study Design

#### Background

This research project is an observational study with a mixed design and will be conducted at the Department of Otolaryngology, Head and Neck Surgery at the Bern University Hospital, Inselspital, Bern, Switzerland. The protocol was designed in accordance with the ethical principles in the Declaration of Helsinki and has been approved by the local institutional review board (reference number: 2017-02037).

#### Participants and Eligibility Criteria

To participate in this study, tinnitus participants have to fulfill the following inclusion criteria: (1) age ≥18 years; (2) subjective tinnitus, that is nonfluctuating; (3) a single-pitched tinnitus perception that is unilateral, bilateral (in both ears), or central (in the head); (4) a difference between the loudness discomfort level (LDL) and minimum masking level (MML) of at least 20 dB; (5) “mild” to “severe” tinnitus, that is, a Tinnitus Handicap Inventory score between 18 and 76 [51]; and (6) sensitivity to RI, that is, a reduction of at least 2 points on a Likert scale of tinnitus loudness change (−5 to +5) directly after the presentation of the acoustic stimulus and for that reduction to be repeated at least 7-10 presentations (see section “Residual Inhibition”).

The exclusion criteria for tinnitus subjects are as follows: (1) LDLs preventing RI stimulation (see section “Tinnitometry”); (2) a history of central nervous system, cardiac, neurologic, psychiatric, or other major diseases or drug abuse, deemed clinically significant at the time of the study by the investigator (Multimedia Appendix 1 for details); (3) moderate or severe depression or generalized anxiety indicated by a Hospital Anxiety and Depression Scale score of at least 11 points on either subscale [52]; and (4) any participant who experiences RI after the 0.5 kHz control stimulus.

Non-tinnitus controls need to meet the following criteria for the study inclusion: (1) age≥18 years; (2) no tinnitus defined by self-reporting; and (3) comparable audiogram to one of the tinnitus subjects, that is, within ±15 dB at each of the frequencies of the extended air conduction hearing thresholds. The values can exceed this threshold at a maximum of 2 frequencies but not at the tinnitus pitch and control frequency.

#### Sample Size

To estimate the appropriate sample size, we used data from a previous study showing statistically significant differences between the PSDs of EEG recordings from tinnitus and nontinnitus participants [53]. The averaged power in the theta band was approximately 15.6 and 14.3 µV^2^ for the tinnitus and nontinnitus subjects, respectively, and the pooled SD was 0.5 µV^2^. A power analysis to test a two-sided hypothesis at a significance level of.05 and a power of 80% was estimated to require a sample size of 39 participants in each group. Up to 50 participants in each group meeting the eligibility criteria after pre-enrollment and the screening session will be recruited.

#### Recruitment

Participants will be recruited through the outpatient clinic and tinnitus consultation at our department. In addition, advertisements will be displayed in public areas and posted on the Web. No staff members from the clinic with a dependency relationship will be recruited. Potential participants who have the willingness and ability to perform all tests required for the study will sign and date an informed consent form before the start of the study procedure.

### Study Procedure

Table 1 provides an overview of the study procedure. During pre-enrollment, a checklist will be filled by potential study participants to assess the tinnitus-associated psychological status (tinnitus subjects only), general health conditions, neurological conditions, and medical history. Potential study participants will then be invited to the screening session to assess the study eligibility. The total duration of the screening session will be 70 minutes for tinnitus subjects and 35 minutes for nontinnitus subjects. Eligible subjects will be invited to the assessment session with a total duration of 150 minutes, including preparation and postassessment cleaning time. The assessment consists of 2 subsessions with 30 minutes each, separated by a 10-minute break. All participants will be asked to avoid the consumption of coffee 5 hours before the assessment session [54].

### Data Collection

#### Infrastructure and Measurement Equipment

All study-specific measurements will be performed in an acoustic chamber (6 m × 4 m × 2 m) certified for clinical audiometry with a broadband reverberation time of approximately 200 ms. The chamber is air-conditioned and provides electromagnetic shielding. For extended audiometry, tinnitometry, and RI assessment, we will use a custom-written script (The MathWorks Inc, v.2017b) with the Psychophysics- Toolbox extensions [55]. Acoustic stimuli will be presented through an external ASIO sound card (Scarlett2i2, FocusRite) and high-definition insert earphones (Triple-Driver, 1MORE Inc). Calibration of the acoustic stimuli (ie, pure tone and third-octave noise) will be confirmed using a head and torso simulator, including 2 ear simulators (Type 4128, Brüel & Kjaer) and an audio analyzer (UPV Audio analyzer DC-250 kHz, Rohde & Schwarz).

**Table 1:** Overview of the study procedure.

Item	Pre-enrollment	Screening session	Assessment session
Medical History	✓	N/A^a^	N/A
Questionnaires (over the Web)	✓	N/A	N/A
Audiometry	N/A	✓	N/A
Tinnitometry	N/A	✓	N/A
Residual Inhibition	N/A	✓	✓
Electroencephalography	N/A	N/A	✓

^a^N/A: not applicable.

#### Medical History and Questionnaires

The medical history contains information about the patients’ health status and the cause of their tinnitus such as the presence of cardiovascular diseases, tinnitus objectivity, signs of otorrhea, and other external or middle ear diseases and drugs that can directly influence the analysis. The following questionnaires will be administered to assess the effects of tinnitus, co- occurring complaints, and health-related quality of life: (1) a general health checklist, aimed at identifying health problems that could affect the brain activity of interest; (2) the Tinnitus Handicap Inventory [56] to assess the severity of tinnitus symptoms; and (3) Hospital Anxiety and Depression Scale [52] to assess depression and anxiety.

#### Audiometry

Standard pure tone audiometry will be performed to assess bone conduction hearing thresholds (in dB hearing level) at 0.5, 0.75, 1, 2, 3, 4, and 6 kHz and air conduction hearing thresholds (in dB hearing level) at 0.125, 0.25, 0.5, 1, 2, 3, 4, 6, and 8 kHz. In addition, an extended assessment of air conduction hearing thresholds (in dB SPL) will be performed at 9, 10, 11, 12, and 13 kHz using a custom-written script and insert earphones.

#### Tinnitometry

Subjects will classify the laterality (ie, unilateral left, unilateral right, bilateral “at both ears,” or central “in the head”) and quality (ie, tonal or noise like) of their tinnitus. The tinnitus pitch will be matched with either pure tone or third-octave noise stimuli ranging from 0.125 to 13 kHz, and the tinnitus loudness at tinnitus pitch (in dB SPL) will be estimated. The MML corresponds to the level of a third-octave noise stimulus (in dB SPL) at which it just renders tinnitus unperceivable. The LDL (in dB SPL) corresponds to the level at which subjects report the stimulus to be uncomfortably loud will be measured.

#### Residual Inhibition

For both ears, the air conduction thresholds for third-octave noise stimuli will be obtained for a stimulus centered at the tinnitus pitch and a 0.5-kHz control frequency. At the tinnitus pitch, the level of the RI stimulus will be specified by adding 20 dB to the MML [57] of the tinnitus ear (in the unilateral case) and by adding 20 dB to the MML of each ear separately (in bilateral or central cases). In the unilateral case, the contralateral ear will be presented with a stimulus level that is specified by adding the RI stimulation sensation level (the difference between the RI stimulus level and the third-octave noise hearing threshold) of the tinnitus ear to its third-octave noise hearing threshold. The third-octave noise control stimulus centered at 0.5 kHz will be presented at the same sensation level as the RI stimulus.

To assess the RI capability, the following procedure will be followed using a 60-second RI stimulus presented through insert earphones. For each of 10 presentations, participants with tinnitus will indicate whether they experience partial or complete suppression of their tinnitus by rating the loudness change on a Likert scale (−5 to +5) immediately after the stimulus presentation. In addition, to obtain time course of their tinnitus change, tinnitus subjects will be asked to continuously rate the loudness change on the Likert scale until their tinnitus has returned to the loudness before the RI stimulus.

#### Electroencephalography

EEG recording will be performed with an active electrode 64-channel biopotential measurement system (ActiveTwo, Biosemi). After selection of a suitable EEG head cap and adjustment of the cap position, electrode gel (Signa Gel, Parker Laboratories, Inc) will be injected, and the electrodes will be attached. The position of the electrodes was selected according to the 10/20 standard scheme for 64 electrodes and 2 additional channels for the active common mode sense and passive-driven right leg electrodes. In addition, the three-dimensional coordinates of all electrodes will be recorded. For electrooculography (EOG) and electromyography (EMG) recording, 8 adhesive surface electrodes will be placed at the head of subjects (left and right outer canthus, right infraorbital and supraorbital, left and right masseter, and left and right mastoid). The offset of each electrode will be checked, measured, and, if required, electrode gel will be added until the offset falls within ±40 mV. Participants will be instructed to use a response box for an automated recording procedure (MATLAB script). Trigger events, such as measurement start, stimulus onsets or offsets, and participants’ responses, will directly recorded by the EEG software (ActiView, Biosemi).

**Figure 1:**
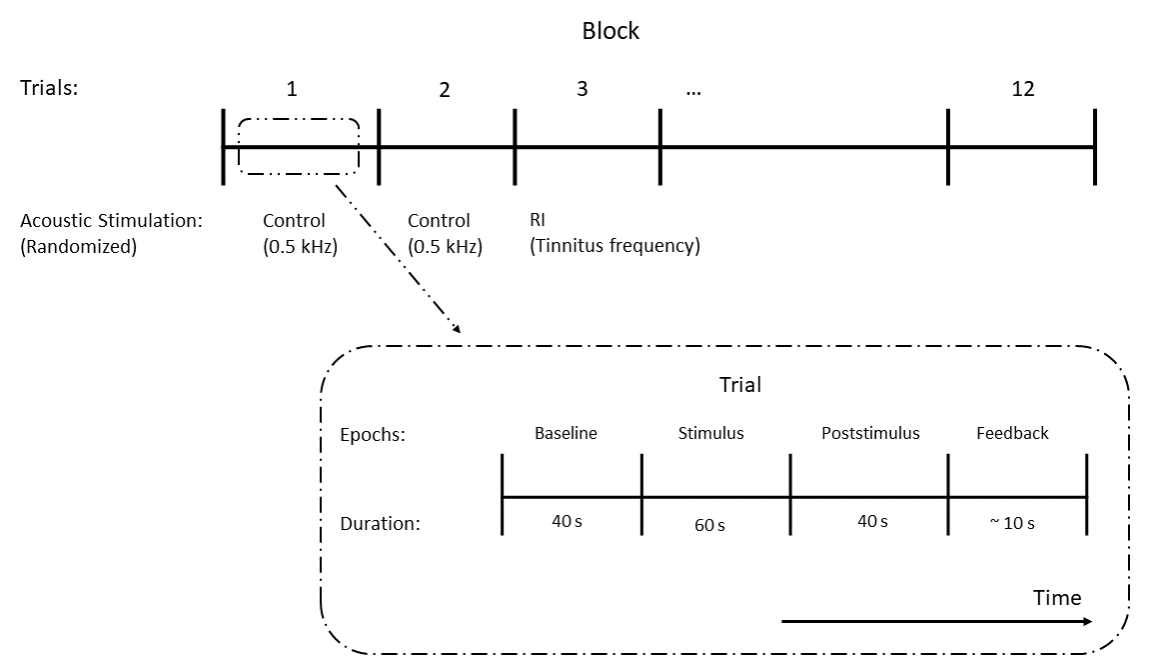
Overview of the electroencephalography recording procedure. RI: residual inhibition.

EEGs will be recorded during 2 blocks consisting of 12 trials each (Figure 1). Each trial consists of 4 epochs—“Baseline,” “Stimulus,” “Poststimulus,” and “Feedback.” The “Baseline” epoch lasts 40 seconds in which spontaneous resting-state EEG is recorded. During the “Stimulus” epoch, the RI and control stimuli will be presented to subjects through the insert earphones for 60 seconds. The RI and control stimuli are played in a randomized sequence to avoid habituation of neural responses.

Subjects are blinded with respect to the order of the stimulation sequence. In the “Poststimulus” epoch, spontaneous resting-state EEG will be recorded for 40 seconds. Tinnitus subjects will be asked to indicate the return of their tinnitus to the normal loudness level by a button press and stay with minimal movement until the end of this epoch. Non-tinnitus controls will be asked to press a button immediately after they perceived a narrow-band stimulus with a central frequency and sensation level same as their matched tinnitus subjects. The stimulus has a duration of 3 seconds and will start 37 seconds after turning off the RI or control stimuli. This process will minimize bias by performing a similar task as tinnitus subjects. In the “Feedback” epoch, EEG recording will be stopped, and participants will be asked to rate the degree of tinnitus loudness change immediately after the stimulus presentation, as well as the change of psychological condition on a scale from −5 to 5 (−5 indicating quieter or feeling less burdened, 0 indicating no change, and +5 indicating louder or feeling more burdened) using the response box. The duration of this epoch is approximately 10 seconds.

Several steps will be taken to suppress or reduce artifacts during EEG recording. The acoustic chamber is electromagnetically shielded. The measurement amplifier is connected by a fiber optic data transfer to the measurement computer outside of the chamber. Before EEG recording is started, light and all power lines will be switched off and no electrical devices, except the battery-powered EEG amplifier, will be active inside the chamber In addition, the chamber will be air-conditioned to avoid contact artifacts of electrodes caused by sweating. During the assessment, participants will be asked to sit relaxed with their eyes closed; they will be instructed to move their body or eyes as little as possible to minimize motion artifacts, especially when making a button press response. To account for muscle and eye artifacts, EOG or EMG are additionally recorded.

### Data Analysis

#### Data Preprocessing

Data preprocessing will be performed with the academic software package Python-MNE [58]. Raw EEG data will be filtered with a zero-phase band-pass filter (0.01-100 Hz). Unpublished results from our preliminary EEG assessments suggest that EEG signals collected in an electromagnetically shielded measurement environment may not need notch filtering for the power line noise removal. The EEG data will be referenced with an average reference. The EEG dataset will be decomposed into independent components using the extended infomax independent component analysis algorithm [59]. The obtained independent components will be visually inspected and compared with EOG and EMG data (using correlation-based analysis). After removing independent components related to artifacts, the EEG data will be reconstructed with the inverse independent component analysis procedure and will be segmented into 1-second epochs.

#### Statistical Analysis

Descriptive statistics will be used to report demographic and baseline characteristics. Quantitative data will be presented as mean, SD, and range (minimum and maximum); qualitative data will be presented as absolute and relative frequencies and, if appropriate, as graphs. We plan to use a linear mixed-effects model to test within-subject and between-group differences in traditional EEG power bands, with the participant group (ie, “tinnitus subject” and “nontinnitus subject”), time-point (ie, “baseline,” “stimulus,” and “poststimulus”), and stimulus type (ie, “RI stimulus” and “control stimulus”) as fixed effects. Subject IDs will be included as a random effect to account for repeated measures. Missing data will not be replaced but treated as “missing” values.

## Results

Ethical approval was obtained in December 2017, and enrollment started in February 2018. The first results are expected in 2019.

## Discussion

Tinnitus is a complex symptom, the pathophysiology of which has perplexed clinicians over the last decades. Recently, neuroimaging techniques with the ability to investigate neural activities have yielded new insights into the tinnitus research [15-20]. The design of this protocol aims to further study abnormal brain oscillations in the coexistence of tinnitus. With the presented within-subject measurement design, we expect minimized data heterogeneity across subjects, which should improve the outcome quality. In addition, high-frequency audiometry, tinnitometry, and RI may provide a more accurate description of tinnitus symptoms. Furthermore, applying the same low-frequency control stimulus to all subjects will help to exclude induced EEG responses caused by external acoustic stimuli. We plan to publish the results in international peer-reviewed open access journals and present at relevant international tinnitus conferences. After the completion of data analysis, anonymized raw or processed data can be made available to interested parties upon request to the corresponding author.

Recently, a data-driven approach using machine learning-based algorithm has been applied to analyze source-localized, resting-state EEG, which was able to classify tinnitus and healthy control subjects with an average accuracy rate of 87.7%, indicating a potential pattern of the neural activity as a cortical signature for tinnitus [60]. Pure data-driven approaches can be used to validate existing theoretical tinnitus models and might advance tinnitus research. Therefore, this protocol was designed to enable structured labeling of EEG data (different conditions of tinnitus perception before and after RI presentation), which can be used for machine learning-based analysis.

## Appendix

Multimedia Appendix 1 Central nervous system, cardiac, neurologic, psychiatric, or other major diseases that are used as exclusion criteria.

## Abbreviations

EEG: electroencephalography
EMG: electromyography
EOG: electrooculography
MEG: magnetoencephalography
MML: minimum masking level
PSD: power spectral density
RI: residual inhibition
LDL: loudness discomfort level
