# Supplementary material for: Association Between Residual Inhibition and Neural Activity in Patients with Tinnitus: Protocol for a Controlled Within- and Between-Subject Comparison Study
Source: JMIR Res Protoc. 2019 Jan 9;8(1):e12270. doi: 10.2196/12270 (PMC6329433; doi:10.2196/12270)
Supplement: Multimedia Appendix 1 [file resprot_v8i1e12270_app1.pdf]

Supplementary table S1. Central nervous system, cardiac, neurologic, psychiatric, or other major diseases that are used as exclusion criteria.

**Medical History**
Traumatic brain injury
Vertigo
Tumor
Epilepsy
Parkinson’s disease
Dementia / Alzheimer
Migraines
Circulatory disorder
Schizophrenia
metabolic disorders
Multiple Sclerosis
Stroke
Heart attack
Heart palpitations
aneurysms
Tremor
Seizures
Diabetes mellitus
Low/high blood pressure
Chronic obstructive pulmonary diseases
Hepatitis
Chronic kidney diseases
